# Plasma and Peritoneal Poly (ADP-Ribose) Polymerase Levels in Patients with Endometriosis

**DOI:** 10.3390/biomedicines10102451

**Published:** 2022-10-01

**Authors:** Joanna Kacperczyk-Bartnik, Paweł Bartnik, Ksawery Goławski, Janusz Sierdziński, Grzegorz Mańka, Mariusz Kiecka, Michał Lipa, Damian Warzecha, Robert Spaczyński, Piotr Piekarski, Beata Banaszewska, Artur Jakimiuk, Tadeusz Issat, Wojciech Rokita, Jakub Młodawski, Maria Szubert, Piotr Sieroszewski, Grzegorz Raba, Kamil Szczupak, Tomasz Kluz, Marek Kluza, Krzysztof Czajkowski, Mirosław Wielgoś, Ewa Koc-Żórawska, Marcin Żórawski, Piotr Laudański

**Affiliations:** 1 II Department of Obstetrics and Gynecology, Medical University of Warsaw, 02-091 Warsaw, Poland; 2 Club 35, Polish Society of Gynecologists and Obstetricians, 53-125 Wrocław, Poland; 3 I Department of Obstetrics and Gynecology, Medical University of Warsaw, 02-091 Warsaw, Poland; 4 Department of Medical Informatics and Telemedicine, Medical University of Warsaw, 00-581 Warsaw, Poland; 5 Angelius Provita Hospital, 40-611 Katowice, Poland; 6 Division of Infertility and Reproductive Endocrinology, Department of Gynecology, Obstetrics and Gynecological Oncology, Poznan University of Medical Sciences, 60-512 Poznan, Poland; 7 Chair and Department of Laboratory Diagnostics, Poznan University of Medical Sciences, 60-512 Poznan, Poland; 8 Department of Obstetrics and Gynecology, Central Clinical Hospital of the Ministry of Interior, 02-507 Warsaw, Poland; 9 Center of Reproductive Health, Institute of Mother and Child in Warsaw, 01-211 Warsaw, Poland; 10 Department of Obstetrics and Gynecology, Institute of Mother and Child in Warsaw, 01-211 Warsaw, Poland; 11 Collegium Medicum, Jan Kochanowski University in Kielce, 25-369 Kielce, Poland; 12 Clinic of Obstetrics and Gynecology, Provincial Combined Hospital in Kielce, 25-736 Kielce, Poland; 13 Department of Gynecology and Obstetrics, Medical University of Lodz, 90-419 Lodz, Poland; 14 Department of Surgical Gynecology and Oncology, Medical University of Lodz, Medical University of Lodz, 90-419 Lodz, Poland; 15 Department of Fetal Medicine and Gynecology, Medical University of Lodz, 90-419 Lodz, Poland; 16 Clinic of Obstetrics and Gynecology in Przemysl, 37-700 Przemysl, Poland; 17 Department of Obstetrics and Gynecology, University of Rzeszow, 35-330 Rzeszow, Poland; 18 Department of Gynecology, Gynecology Oncology and Obstetrics, Institute of Medical Sciences, Medical College of Rzeszow University, 35-310 Rzeszow, Poland; 19 II Department of Nephrology and Hypertension with Dialysis Unit, Medical University of Bialystok, 15-089 Bialystok, Poland; 20 Department of Clinical Medicine, Medical University of Bialystok, 15-089 Bialystok, Poland; 21 OVIklinika Infertility Center, 01-377 Warsaw, Poland

**Keywords:** endometriosis, ELISA, infertility, PARP, peritoneal fluid, plasma

## Abstract

The evidence of poly (ADP-ribose) polymerase (PARP) association with the immune response could be coherent with the immunological theory of endometriosis and suggests the possibility of a new research direction. The aim of the study was to evaluate the levels of PARP in plasma and peritoneal fluid of patients with and without endometriosis. It was a multicenter, cross-sectional study. Plasma and peritoneal fluid samples were collected from patients with and without endometriosis during planned laparoscopic procedures in eight clinical centers. In total, 84 samples of plasma and 84 samples of the peritoneal fluid were included in the final analyses. Double-antibody sandwich enzyme-linked immunosorbent assay was performed in order to assess levels of PARP in collected samples. No statistically significant differences regarding the detected levels of PARP in plasma and peritoneal fluid comparing patients with and without endometriosis were observed. Patients with a history of infertility had significantly higher plasma PARP concentrations (*p* = 0.04). We have not observed the potential role of PARP concentration levels in plasma nor peritoneal fluid as an endometriosis biomarker. We have determined an association between a higher plasma PARP concentration and a history of infertility.

## 1. Introduction

Endometriosis is a chronic gynecological disorder associated with pelvic pain and infertility. It is characterized by the presence of uterine endometrial tissue outside of the uterus—on the pelvic peritoneum, on the ovaries, in the rectovaginal septum, and rarely in the pericardium, pleura, and even brain. Pelvic endometriosis affects 6–10% of the general female population. Among women with pain and/or infertility the prevalence reaches 50% [1,2]. Multiple theories present the role of different pathogenic factors involved in the development of endometriosis: retrograde menstruation, metaplasia, hormones, oxidative stress and inflammation, immune dysfunction, apoptosis suppression, genetic factors and stem cell activity [3,4,5,6,7,8,9,10]. Complex etiology of the disease leads to a diagnostic delay exceeding seven years [11].

The immune system is also believed to be involved in the pathogenesis of endometriosis [12,13]. Inadequate immune surveillance in the peritoneum may be associated with the disorder. It is reported in the literature that patients with endometriosis are characterized by activation of peritoneal macrophages with increased cytokine production, although there is decreased phagocytic activity [12,14]. Compromised natural-killer-cell activity in the peritoneal fluid can lead to decreased surveillance of ectopic tissue [15].

Women with endometriosis are at increased risk of developing malignant tumors of the pelvis [16,17]. The history of endometriosis is present in a significantly higher proportion of women undergoing surgery for endometroid, clear-cell, and mixed subtypes of ovarian cancers than in women with serous, mucinous, and other subtypes of ovarian malignancies [18]. Several reports suggest that women with endometriosis also have an increased risk of developing other types of cancers [19,20,21,22].

Certain features and mechanisms of endometriosis contribute to its similarity to oncological diseases. Cellular metaplasia, tissue migration, angiogenesis, and organ infiltration are both characteristic for endometriosis and different stages of cancer. Processes investigated in reference to progression and treatment of oncological diseases may also be worth examining in cases of endometriosis.

Poly (ADP-ribose) polymerase-1 (PARP-1) was the first identified member of the PARP family, which now includes 18 different proteins [23]. PARP-1 accounts for more than 90% of cellular PARP activity. The main role of PARP-1 is to catalyze the polymerization of ADP-ribose units—derived from the ADP donor NAD+—resulting in the attachment of PAR polymers to itself or to other proteins [24]. The activity of PARP-1 is stimulated by various factors, including DNA damage [25,26]. The generation of PAR follows metabolic, oxidative, oncogenic or genotoxic stress.

A large number of studies revealed that PARP-1 interacts with other transcription factors, mainly interferon regulatory factor 1 (IRF1) and participates in cell defense against viral and bacterial infections. PARP-1 plays a role in the development of innate and adaptive responses as well as immune cell differentiation [27,28].

This evidence of PARP-1 association with the immune response could be coherent with the immunological theory of endometriosis and suggests the possibility of another direction in the PARP-1 and endometriosis research. Multiple studies focused on evaluation of different pro- and anti-inflammatory factors in specimens collected from endometriosis patients; however, data on PARP concentrations in plasma and peritoneal fluid is lacking [4,5,6,7,9,29,30].

The primary aim of the study was to evaluate the levels of PARP in plasma and peritoneal fluid of patients with and without endometriosis. The secondary aim of the study was to examine the levels of PARP in patients with and without the history of infertility.

## 2. Materials and Methods

This was a multicenter, cross-sectional study. Plasma and peritoneal fluid samples were collected from 50 patients with (study group) and 48 without (control group) endometriosis during planned surgical procedures in eight Polish clinical centers between 2018 and 2019 (project number: 6/6/4/1/NPZ/2017/1210/13522, financed by the Polish Ministry of Health).

Recruited patients were between 18 and 40 years old, qualified for planned laparoscopic procedures due to non-malignant indications: infertility, chronic pelvic pain syndrome, ovarian cysts, endometriosis. Infertility was defined as the inability to achieve pregnancy during twelve months or more of regular unprotected sexual intercourse for women younger than 35 years old and 6 months or more for the rest of women [31]. Exclusion criteria were: irregular menstruation, hormonal treatment within three months before the surgery, pelvic inflammatory disease, uterine fibroids, polycystic ovary syndrome, autoimmune comorbidities, malignancies, and any previous history of surgical treatment. Each patient was evaluated on the basis of the revised American Fertility Society classification of endometriosis together with histology examination of the collected specimen [32]. Patients without visible endometriosis during laparoscopy were recruited to the control group. Based on inspection during laparoscopy, patients with endometriosis were allocated to the adequate endometriosis stage subgroup (I–IV). Patients were fasting between 6–12 h before the surgery. Additionally, prior to the surgery blood samples were collected in ethylenediaminetetraacetic acid (EDTA) 10 mL tubes (Sarstedt) to obtain specimen for plasma PARP level evaluation. Material collection did not influence medical management of patients and was performed in accordance with the Declaration of Helsinki. Peritoneal fluid was collected via Veress needle aspiration under direct visual inspection in the beginning of the laparoscopy in order to avoid contamination with blood. The procedure was performed each time in accordance with the Endometriosis Phenome and Biobanking Harmonisation Project standard operating procedures [33]. Aspirated peritoneal fluid was spun at 1000× *g* for 10 min at 4 °C. The supernatant was transferred to a fresh 10 mL tube (Sarstedt). The time lapse between sample collection (both peritoneal fluid and plasma) and processing was less than 45 min. All centers centrifuged blood samples at 2500× *g* for 10 min at 4 °C. Specimen samples were stored at −80 °C.

Double-antibody sandwich enzyme-linked immunosorbent assay (ELISA) was performed in order to assess levels of PARP in collected plasma and peritoneal fluid samples. ELISA is a quantitative method which has been used for decades for detection and quantification of specific substances [34,35]. It has also been widely performed in order to detect PARP in human biological samples [36,37,38]. Human PARP ELISA kits (SunRedBio, Shanghai, China) were used with the sensitivity of 7.282 ng/L and assay range 8 ng/L–2000 ng/L. Quantitative PARP levels analyses were financed by an internal grant from the Medical University of Warsaw (project number 1W51/1/M/MB/N/20). Study protocol was approved by the Institutional Review Board at the Medical University of Warsaw (approval number AKBE/131/2020).

Outliers were detected and then excluded using classic statistical domain based on interquartile range. After exclusion of the outlier results, 84 samples of plasma (47 from patients with and 37 from patients without endometriosis) and 84 samples of the peritoneal fluid (48 from patients with and 36 from patients without endometriosis) were included in the final analyses.

Statistical analysis was performed with SAS v. 9.4 (SAS Institute, Cary, NC, USA) and Statistica v. 13.3 software (StatSoft Inc., Kraków, Poland). The groups were compared by Chi-square test for categorical variables. Mann–Whitney U test and Student’s *t*-test were performed for continuous variables depending on the distribution of variables after testing for normal distribution using the Shapiro–Wilk test. The level of statistical significance was set at *p* < 0.05.

## 3. Results

Table 1 and Table 2 show characteristics and results of PARP levels in the study and control groups. No statistically significant differences regarding the detected levels of PARP in the plasma and in the peritoneal fluid comparing patients with and without endometriosis were observed.

Table 3 presents the results of PARP levels comparison between patients with different endometriosis stages and women without endometriosis. There was a higher level of plasma mean (2339.5 vs. 960.33 ng/L) and median (830.35 vs. 243.1 ng/L) PARP concentrations in patients with stage I and II compared to patients with stage III and IV but the difference was not statistically significant (*p* = 0.12).

Additional analyses shown in Table 4 examined the association between PARP levels and infertility. Patients with a history of infertility had significantly higher plasma PARP concentrations than patients without infertility (*p* = 0.04). A tendency towards higher peritoneal mean (817.12 vs. 563.8 ng/L) and median (371.5 vs. 267.8 ng/L) PARP levels were characteristic for the history of infertility, but no statistically significant differences regarding PARP levels in the peritoneal fluid comparing patients with and without infertility were detected (*p* = 0.057). We also observed higher plasma mean (2471.54 vs. 778.94 ng/L) and median (1062 vs. 387.5 ng/L) PARP concentrations in patients with primary infertility compared to patients with secondary infertility, yet the difference was not significant (*p* = 0.49).

Comparison of plasma and peritoneal fluid PARP concentrations within the endometriosis group did not show any statistically significant differences between women with and without infertility (Table 4).

## 4. Discussion

The main finding of our study was a detected difference in plasma PARP concentrations of patients with infertility compared to women without infertility. A few reports available in the literature concern the PARP expression and endometrial receptivity. Joshi et al. examined the role of PARP-1 in embryo implantation and uterine decidualization in mice [39]. Authors observed that PARP-1 was upregulated and its expression in cytosol was elevated at implantation sites during the pre-implantation period. During the post-implantation stage there was a decrease in PARP-1 levels. The level of nuclear PARP-1 transcript fraction was increased both at implantation and non-implantation sites during the pre- and peri-implantation stages. During the post-implantation stage, PARP-1 expression was downregulated with lower levels in the non-implantation sites than in the implantation sites.

In the same study by Joshi et al. authors also examined PARP-1 expression during the uterine decidualization [39]. Significantly higher PARP-1 expression was observed in the decidualized uterus compared to non-decidualized uterine tissue. The functional role of PARP-1 in the implantation process was confirmed by an observed reduced number of implantation sites and blastocyst numbers following intra-luminal administration of PARP-1 inhibitor during the pre-implantation stage. The authors also detected associations between PARP-1 expression and hormonal exposure. Progesterone administration resulted in lower level of PARP-1, whereas during estrogen supplementation PARP-1 levels were significantly higher [39].

Soni et al. used a mouse model in order to evaluate the role of PARP-2 in the embryo implantation process [40]. The authors determined PARP-2 expression in endometrial tissue during the window of implantation period. It was observed that PARP-2 in the cytosol was upregulated in the pre-, late pre- and peri-implantation stages. In the advanced stage, the expression of PARP-2 in the nuclear compartment was elevated in the implantation sites only. The authors also confirmed the influence of progesterone and estrogen on PARP-2 expression and resulting endometrial receptivity adjustments. Similarly to the previous study about PARP-1, the authors observed increased PARP-2 expression in a decidualized mice uterus as well as a decreased implantation rate associated with PARP-2 inhibition. What is more, PARP-2 expression was detected not only in the uterine tissue throughout the window of implantation, but also in mice embryos [40].

The role of PARPs and poly(ADP-ribosyl)ation in the pre-implantation development and epigenetic remodeling of mice zygotes was also reported by Imamura et al. [41]. The authors observed transient and reversible upregulation of poly(ADP-ribosyl)ation shortly after oocyte fertilization. The role of PARP-1 and PARP-2 in the embryogenesis was also confirmed in a study by Ménissier de Murcia et al. in which the authors observed that parp-1-/-parp2-/- double mutant mice embryos are not viable and die at the onset of gastrulation [42].

Our negative results regarding no observation of significant differences in PARP plasma and peritoneal fluid levels between patients with and without endometriosis and between patients with different endometriosis stages do not exclude the possibility of a PARP role in the endometriosis pathogenesis. Barreta et al. detected similar PARP-1 expression levels in endometriosis-related benign ovarian lesions compared to specimens obtained from endometriosis-associated ovarian carcinomas [43]. In a study by Talebi et al. it was observed that rats with induced endometriosis had higher expression of pro-PARP in comparison to rats exposed to rutin or vitamin C for possible endometriosis treatment [44]. Ekici et al. investigated PARP-1 expression in neutrophils of patients with endometriosis and the impact of cabergoline administration on its levels [45]. The authors observed that PARP-1 expression was higher in the neutrophils of endometriosis patients compared to healthy controls. What is more, PARP-1 expression was reduced in the subgroup of women with endometriosis and cabergoline intake. In a study by Yang et al. the authors propose a novel possible mechanism of endometriosis pathophysiology involving PGE2-induced apoptosis suppression by upregulation of Cav1.3 expression resulting in decreased cleaved PARP and caspase 3 levels [46].

One of the limitations of our research is the size of the study group, which could be the reason of obtaining non-significant results regarding the difference in PARP concentrations in the peritoneal fluid of patients with and without infertility. Further cooperation and investigations within the multicenter endometriosis working group in Poland are planned in the near future.

The second limitation of the study concerns the differences in the menstrual cycle day during sample collection of patients with (on average on the 14th day) and without endometriosis (on average on the 11th day) as it is documented that hormonal levels affect PARP expression [39,40]. What is more, in a study by Ghabreau et al. the authors examined expression levels of PARP-1 in normal endometrial epithelium depending on the menstrual cycle phase [47]. It was reported that PARP-1 expression was high in the proliferative phase with the highest level during the late proliferative phase and significantly decreased in the secretory phase (*p* = 0.0002). Authors also observed that in most cases (except non-endometrioid carcinomas) PARP-1 expression positively correlated with progesterone receptor expression (*p* < 0.0001) [47].

It is also worth emphasizing that in our relatively homogenous group of patients high fluctuations of PARP levels were observed. This suggests that multiple factors can be responsible for detectable plasma and peritoneal fluid PARP levels and questions the possibility of using liquid tissue PARP concentration as biomarkers.

The strong point of the study is the analysis of PARP concentrations in samples collected from the human population of women diagnosed with endometriosis and with infertility as the majority of published reports about PARPs role in fertility present data obtained using rodent models.

## 5. Conclusions

We have not observed the potential role of PARP concentration levels in plasma nor peritoneal fluid as an endometriosis biomarker. We have determined the association between higher plasma PARP concentration and a history of infertility.

## Figures and Tables

**Table 1:** Group characteristics and PARP levels detected in plasma of patients with and without endometriosis.

Feature	Patients with Endometriosis (*n* = 47)	Patients without Endometriosis (*n* = 37)	*p*
Age [years]	31.79 (SD ± 5.24)	31.49 (SD ± 5.77)	0.96
Day of menstrual cycle	14.8 (SD ± 5.97)	11.68 (SD ± 5.58)	0.02
Stage I endometriosis	13 (28%)	Not applicable	-
Stage II endometriosis	7 (15%)	Not applicable	-
Stage III endometriosis	15 (32%)	Not applicable	-
Stage IV endometriosis	10 (21%)	Not applicable	-
Infertility	28 (60%)	19 (51%)	0.51
Primary infertility	23 (49%)	13 (35%)	0.27
Secondary infertility	5 (11%)	6 (16%)	0.52
Endometrial ovarian cysts	28 (60%)	Not applicable	-
Mean PARP concentration [ng/L]	1544.06 (SD ± 3455.73)	1554.92 (SD ± 3250.36)	0.73
Median PARP concentration [ng/L]	442.7 (55.55–20360)	451 (27.17–15390)	

**Table 2:** Group characteristics and PARP levels detected in peritoneal fluid of patients with and without endometriosis.

Feature	Patients with Endometriosis (*n* = 48)	Patients without Endometriosis (*n* = 36)	*p*
Age [years]	31.85 (SD ± 4.82)	31.2 (SD ± 6.02)	0.98
Day of menstrual cycle	14.81 (SD ± 6.19)	11.56 (SD ± 5.72)	0.02
Stage I endometriosis	15 (31%)	Not applicable	-
Stage II endometriosis	6 (13%)	Not applicable	-
Stage III endometriosis	17 (35%)	Not applicable	-
Stage IV endometriosis	9 (19%)	Not applicable	-
Infertility	19 (40%)	21 (58%)	0.12
Primary infertility	18 (36%)	17 (47%)	0.38
Secondary infertility	1 (2%)	4 (11%)	0.19
Endometrial ovarian cysts	27 (56%)	Not applicable	-
Mean PARP concentration [ng/L]	760.2 (SD ± 676.78)	646.73 (SD ± 647.56)	0.61
Median PARP concentration [ng/L]	451 (12.55–2219)	294.15 (126.6–2391)	

**Table 3:** Comparison of plasma and peritoneal fluid PARP levels depending on endometriosis stages.

**Plasma PARP Concentrations [ng/L]**			
	**Mean (±SD)**	**Median**	*p*
Patients with endometriosis stages I and II (*n* = 20)	2339.5 (±4746.14)	830.35 (63.63–20,360)	0.12
Patients with endometriosis stages III and IV (*n* = 25)	960.33 (±2022.69)	243.1 (55.55–10,180)	
Patients with endometriosis stages I and II (*n* = 20)	2339.5 (±4746.14)	830.35 (63.63–20,360)	0.44
Patients without endometriosis (*n* = 37)	1554.92 (± 3250.36)	451 (27.17–15,390)	
Patients with endometriosis stages III and IV (*n* = 25)	960.33 (±2022.69)	243.1 (55.55–10,180)	0.23
Patients without endometriosis (*n* = 37)	1554.92 (± 3250.36)	451 (27.17–15,390)	
**Peritoneal fluid PARP concentrations [ng/L]**			
Patients with endometriosis stages I and II (*n* = 21)	861.13 (±725)	646.6 (12.55–2391)	0.77
Patients with endometriosis stages III and IV (*n* = 26)	700.18 (±644.82)	451 (109.5–2219)	
Patients with endometriosis stages I and II (*n* = 21)	861.13 (±725)	646.6 (12.55–2391)	0.52
Patients without endometriosis (*n* = 36)	646.73 (± 647.56)	294.15 (126.6–2391)	
Patients with endometriosis stages III and IV (*n* = 26)	700.18 (±644.82)	451 (109.5–2219)	0.7
Patients without endometriosis (*n* = 36)	646.73 (± 647.56)	294.15 (126.6–2391)	

**Table 4:** PARP levels detected in plasma and peritoneal fluid of patients with and without infertility.

**Plasma PARP Concentrations [ng/L]**			
	**Mean (±SD)**	**Median**	*p*
Patients with infertility (*n* = 47)	2075.4 (±4149.94)	1047 (55.55–20,360)	0.04
Patients without infertility (*n* = 37)	879.98 (±1731.04)	213.2 (27.17–10,180)	
Patients with primary infertility (*n* = 36)	2471.54 (±4670.41)	1062 (55.55–20,360)	0.49
Patients with secondary infertility (*n* = 11)	778.94 (±680.48)	387.5 (106.7–2280)	
Endometriosis patients with infertility (*n* = 28)	1897.71 (±4065.21)	702.35 (55.55–20,360)	0.2
Endometriosis patients without infertility (*n* = 19)	1022.88 (±2290.97)	213.2 (98.95–10,180)	
Non-endometriosis patients with infertility (*n* = 19)	2337.25 (±4370.53)	1076 (90.71–15,390)	0.16
Non-endometriosis patients without infertility (*n* = 18)	729.13 (±861)	208.95 (27.17–2357)	
**Peritoneal fluid PARP concentrations [ng/L]**			
Patients with infertility (*n* = 49)	817.12 (±701.53)	371.5 (12.55–2391)	0.057
Patients without infertility (*n* = 35)	563.8 (±582.48)	267.8 (126.6–2219)	
Patients with primary infertility (*n* = 39)	833.96 (±700.86)	371.5 (12.55–2219)	0.74
Patients with secondary infertility (*n* = 10)	751.46 (±738.13)	393.05 (170.3–2391)	
Endometriosis patients with infertility (*n* = 17)	536.91 (±522.44)	278.1 (12.55–2219)	0.1
Endometriosis patients without infertility (*n* = 31)	882.65 (±726.81)	646.6 (126.9–1906)	
Non-endometriosis patients with infertility (*n* = 18)	704.26 (±660.36)	311.5 (170.3–2391)	0.26
Non-endometriosis patients without infertility (*n* = 36)	646.73 (±647.56)	294.15 (126.6–2219)	

## Data Availability

The data presented in this study are available on request from the corresponding author. The data are not publicly available due to privacy.
